# Supplementary material for: Effectiveness of in-group versus individually administered pain neuroscience education on clinical and psychosocial outcomes in patients with chronic low back pain: randomized controlled study protocol
Source: PeerJ. 2024 May 31;12:e17507. doi: 10.7717/peerj.17507 (PMC11146333; doi:10.7717/peerj.17507)
Supplement: Supplemental Information 2 [file peerj-12-17507-s002.pdf]

ClinicalTrials.gov Protocol Registration and Results System (PRS) Receipt
Release Date: July 19, 2023

ClinicalTrials.gov ID: NCT05953454

Study Identification

Unique Protocol ID: USantoTomasChile

Brief Title: Effectiveness of Pain Neuroscience Education on Clinical and Psychosocial Variables in Chronic Low Back Pain

Official Title: Effectiveness of Group Versus Individual Pain Neuroscience Education on Clinical and Psychosocial Outcomes in Patients With Chronic Low Back Pain: Protocol of a Randomized Controlled Trial

Secondary IDs:

Study Status

Record Verification: July 2023

Overall Status: Not yet recruiting

Study Start: April 1, 2024 [Anticipated]

Primary Completion: December 1, 2024 [Anticipated]

Study Completion: December 30, 2024 [Anticipated]

Sponsor/Collaborators

Sponsor: Universidad Santo Tomas, Chile

Responsible Party: Principal Investigator
Investigator: Joaquín Ignacio Salazar Méndez [jisalazarmendez]
Official Title: adjunct teacher
Affiliation: Universidad Santo Tomas, Chile

Collaborators:

Oversight

U.S. FDA-regulated Drug: No

U.S. FDA-regulated Device: No

U.S. FDA IND/IDE: No

Human Subjects Review: Board Status: Not yet submitted
Board Name: Comité de Ética macrozona centro-sur, Universidad Santo Tomás
Board Affiliation: Universidad Santo Tomás, Chile
Phone: (41) 2536628
Email: comitedeeticacs@santotomas.cl
Address:

Data Monitoring: No

FDA Regulated Intervention: No

Study Description

Brief Summary: An educational intervention on the neurophysiology of chronic pain will be provided. The content of the intervention will be identical in the experimental groups (group and individual). The intervention has an active educational approach based on reconceptualizing the maladaptive beliefs that influence the fear-avoidance behavior of the participants through updated contents of the neuroscience of pain.

The effects of the intervention will be compared between the groups and the influence of the social determinants of health on the effects will also be determined.

The investigators hypothesize that there will be significant differences in favor of the group intervention group over the individual intervention groups. Furthermore, the effects will be influenced by the social determinants of health in both experimental groups.

Detailed Description:

Conditions

Conditions: Chronic Low-back Pain

Keywords: pain neuroscience education
physical therapy
chronic pain

Study Design

Study Type: Interventional

Primary Purpose: Treatment

Study Phase: N/A

Interventional Study Model: Parallel Assignment

Number of Arms: 3

Masking: Double (Participant, Outcomes Assessor)

Allocation: Randomized

Enrollment: 69 [Anticipated]

Arms and Interventions

Arms	Assigned Interventions
Experimental: Group Pain Neuroscience Education<br>A single face-to-face group session of approximately 60-80 minutes provided through active participation. Five key domains will be structured from the Fear and Belief Avoidance Questionnaire that will serve as a guide for the sessions through a Powerpoint presentation. In addition, participants will be	Pain neuroscience Education<br>A pain neuroscience education session geared towards fear-avoidance beliefs

Arms	Assigned Interventions
encouraged to be active by walking for 20-30 minutes 3-5 times a week and will be taught an exercise to improve transverse abdominis activation (abdominal corset or follow-through).<br><br>With the main ones, a brochure will be delivered and informative capsules will be made to which the participants will have access (5 videos of 15 minutes, one per domain). Participants will be instructed to record on a calendar the days they read the brochure and/or reviewed the information capsules to assess treatment compliance and for each domain invent a metaphor or script for how they would explain it to another person. This activity must be delivered in the second evaluation.	
Experimental: Individual Pain Neuroscience Education<br>A single one-on-one face-to-face session of approximately 60-80 minutes provided through active participation. Five key domains will be structured from the Fear and Belief Avoidance Questionnaire that will serve as a guide for the sessions through a Powerpoint presentation. In addition, participants will be encouraged to be active by walking for 20-30 minutes 3-5 times a week and will be taught an exercise to improve transverse abdominis activation (abdominal corset or follow-through).<br><br>With the main ones, a brochure will be delivered and informative capsules will be made to which the participants will have access (5 videos of 15 minutes, one per domain). Participants will be instructed to record on a calendar the days they read the brochure and/or reviewed the information capsules to assess treatment compliance and for each domain invent a metaphor or script for how they would explain it to another person. This activity must be delivered in the second evaluation.	Pain neuroscience Education<br>A pain neuroscience education session geared towards fear-avoidance beliefs
No Intervention: Control<br>no intervention	

Outcome Measures

Primary Outcome Measure:

1. Changes in Fear avoidance beliefs
   The Fear Avoidance Beliefs Questionnaire consists of two subscales: (1) a 7-item work subscale (FABQ-W) and (2) a 4-item physical activity subscale (FABQ-P). Both subscales score on a Likert-type scale from 0 to 6 points on each item. Higher scores indicate higher levels of fear-avoidance beliefs.

   [Time Frame: baseline, 1-week post-intervention, and 4 -weeks post-intervention]
2. changes in pressure pain sensitivity
   An algometer will be used to measure pressure pain sensitivity (SPD), which is defined as the amount of applied pressure required for a subject to report the onset of pain sensation. It will be applied three times for each moment of evaluation and the average of the three applications will be considered. The unit of measure kg/cm2/s will be used.

   [Time Frame: baseline, 1-week post-intervention, and 4 -weeks post-intervention]

Secondary Outcome Measure:

3. Changes in Pain Self-efficacy

Cain self-efficacy questionnaire (PSEQ) consists of 10 items rated on a 7-point Likert scale from 0 ("not at all sure") to 6 ("very sure"). Higher scores indicate stronger self-efficacy beliefs, while low scores indicate a subject more focused on their pain.

[Time Frame: baseline, 1-week post-intervention, and 4 -weeks post-intervention]

4. Changes in Catastrophizing
   Pain Catastrophizing Scale (PCS) consists of 13 items on a 5-point Likert scale ranging from (0) never to (4) all the time. Higher scores indicate more catastrophic thoughts.

   [Time Frame: baseline, 1-week post-intervention, and 4 -weeks post-intervention]
5. Changes in Pain intensity
   Numerical Rating Scale (NRS). It consists of a number line from 0 to 10. Higher scores indicate greater intensity of pain.

   [Time Frame: baseline, 1-week post-intervention, and 4 -weeks post-intervention]
6. Treatment expectation
   The treatment expectation questionnaire (TEX-Q). This questionnaire consists of 15 questions with a Likert scale of 0-10. higher scores indicate a better expectation of treatment.

   [Time Frame: baseline]

Other Pre-specified Outcome Measures:

7. Employment status
   employed versus unemployed

   [Time Frame: baseline]
8. Educational level
   participants were assigned to the lower educational level if they had not completed secondary education and to the higher educational level if they had completed secondary education or university studies

   [Time Frame: baseline]
9. economic income
   individual monthly taxable income

   [Time Frame: baseline]

Eligibility

Minimum Age: 45 Years

Maximum Age: 60 Years

Sex: All

Gender Based: No

Accepts Healthy Volunteers: No

Criteria: Inclusion Criteria:

- non-specific low back pain ≥ 3 months without compromise of any lower limb.
- average pain intensity ≥ 3/10 and ≤8/10 (according to the 0-10 numerical rating scale [NRS]) in the last month.

Exclusion Criteria:

- psychiatric, neurological or oncological diseases.
- operated of some lumbar pathology
- chronic low back pain due to a specific cause (lumbar stenosis, herniated disc, spinal deformity, fracture, spondylosis)

- have received any modality of active or passive physical therapy for pain in the last two months,
- previous experiences with PNE

Contacts/Locations

Central Contact Person: Joaquín I Salazar, MSc
Telephone: +569 73785287
Email: jsalazar13@santotomas.cl

Central Contact Backup:

Study Officials: Joaquín I Salazar, MSc
Study Principal Investigator
Universidad Santo Tomás

Locations:

IPDSharing

Plan to Share IPD: Yes
all collected IPD, all IPD that underlie results in a publication

Supporting Information:
Study Protocol
Statistical Analysis Plan (SAP)
Informed Consent Form (ICF)
Clinical Study Report (CSR)

Time Frame:
from 6 months after publication. They will be available for one year.

Access Criteria:
will be provided to any researcher who requires it via email

URL:

References

Citations:

Links:

Available IPD/Information:

U.S. National Library of Medicine | U.S. National Institutes of Health | U.S. Department of Health & Human Services
